# Effects of Minor Zn Dopants in Sn-10Bi Solder on Interfacial Reaction and Shear Properties of Solder on Ni/Au Surface Finish

**DOI:** 10.3390/ma17174364

**Published:** 2024-09-03

**Authors:** Sijin Li, Junxian Zhu, Huiling Zhou, Mingqing Liao, Fengjiang Wang, Jian Chen

**Affiliations:** 1 School of Materials Science and Engineering, Jiangsu University of Science and Technology, Zhenjiang 212000, Chinamingqing_liao@just.edu.cn (M.L.); fjwang@just.edu.cn (F.W.); 2 Department of Chemistry and Surface Science Western, University of Western Ontario, London, ON N6A 5B7, Canada

**Keywords:** Sn-Bi solder, intermetallic compounds, Ni/Au surface finish, shear strength, Zn dopant

## Abstract

Sn-10Bi low-bismuth-content solder alloy provides a potential alternative to the currently used Sn-Ag-Cu series due to its lower cost, excellent ductility, and strengthening resulting from the Bi solid solution and precipitation. This study primarily investigates the interfacial evolution and shear strength characteristics of Sn-10Bi joints on a Ni/Au surface finish during the as-soldered and subsequent isothermal aging processes. To improve the joint performance, a 0.2 or 0.5 wt.% dopant of Zn was incorporated into Sn-10Bi solder. The findings demonstrated that a 0.2 or 0.5 wt.% Zn dopant altered the composition of the intermetallic compound (IMC) formed at the interface between the solder and Ni/Au surface finish from Ni_3_Sn_4_ to Ni_3_(Sn, Zn)_4_. The occurrence of this transformation is attributed to the diffusion of Zn atoms into the Ni_3_Sn_4_ lattice, resulting in the substitution of a portion of the Sn atoms by Zn atoms, thereby forming the Ni_3_(Sn, Zn)_4_ IMC during the soldering process, which was also verified by calculations based on first principles. Furthermore, a 0.2 or 0.5 wt.% Zn dopant in Sn-10Bi significantly inhibited the Ni_3_(Sn, Zn)_4_ growth after both the soldering and thermal aging processes. Zn addition can enhance the shear strength of solder joints irrespective of the as-soldered or aging condition. The fracture mode was determined by the aging durations—with the brittle mode occurring for as-soldered joints, the ductile mode occurring for aged joints after 10 days, and again the brittle mode for joints after 40 days of aging.

## 1. Introduction

In the electronics industry, the application of Pb-free solder alloys is restricted by the RoHS (Restriction of Hazardous Substances) directive and health concerns because Sn-37Pb eutectic solder includes Pb, which is harmful to health and pollutes groundwater [1]. Sn-Ag-Cu series like SAC105, SAC305, SAC405, SAC387, and SAC396 are most frequently applied due to their excellent wettability and solder joint reliability [2]. However, the higher melting point of Sn-Ag-Cu series (217~221 °C) makes it very difficult to use soldering-temperature-sensitive components, which have lower processing temperature requirements. Sn-Bi solder presents a eutectic reaction in the Sn-58Bi composition at the eutectic temperature of 138 °C and has gradually been accepted for low-temperature soldering conditions [3,4]. However, because there exists a considerable amount of Bi in Sn-Bi eutectic solder, and Bi is very brittle, Sn-58Bi solder often leads to reliability issues, including a reduction in performance and interfacial failure [5]. Bi atoms can be easily dissolved in the Sn matrix with the maximum solubility at around 22 wt.% to form a solid solution, which can provide a potential application with low-Bi-content Sn-Bi solder. This solder includes a microstructure with β-Sn as the primary phase and Bi atoms as the solid-solution-strengthening phase, which helps it to provide the excellent mechanical properties, including enhanced tensile and fracture strength [6,7,8]. There have been many studies on low-Bi-content Sn-Bi alloys in recent years. Guo et al. [8] compared Sn-10Bi solder with Sn-Ag-Cu solder and found that Sn-10Bi solder presented higher creep resistance and tensile strength. Wang et al. [9] compared Sn-15Bi solder with Sn-Pb eutectic solder and found that the Sn-15Bi solder presented a higher tensile strength for solder alloy and a higher shear strength for ball solder joints. Kang et al. [10] studied IMC growth in a Sn-Bi solid solution solder under high-temperature aging conditions. Therefore, low-Bi-content Sn-Bi solder provides a potential low-cost replacement for Sn-Ag-Cu solder.

During soldering, an interfacial intermetallic compound (IMC) forms at the interface between the molten solder and the metallic surfaces being joined. This compound has mechanical adhesion, electrical conductivity, and wetting properties [11]. However, the inherent frangibility and the rapid growth of IMC layers at the interface deteriorate the reliability of the solder joint [12,13,14,15]. Especially in Sn-Bi solder, the increase in Bi content in the solder obviously promotes the growth of the interfacial IMC layer [16]. Alloying Sn-Bi solder with minor elements is the best method for controlling the microstructure of solder matrix and the interfacial reaction of IMC layer of solder joints. For example, in Sn-5Bi solder, Zhang et al. [17] introduced minor Sb to realize the precipitation-strengthening effect and minor In to enhance the solid-solution-strengthening effect. Both of these elements were found to be helpful for improving the shear strength and ductility of Sn-5Bi solder joints. Han et al. [18] adopted minor Cu and Cr in Sn-10Bi solder to improve its wettability and the depression effect on IMC growth. In particular, the addition of minor Zn into solder has received much attention in the development of Pb-free solders. Lin et al. introduced a minor Zn dopant into Sn-15Bi solder to depress the interfacial growth in the solder on Cu [19]. Xu et al. incorporated 0.5Zn into Sn-Bi-In solder and observed a higher dissolution of Zn in the (In, Bi)Sn_4_ phase, which enhanced the strength of the solder and refined the solder microstructure [20]. Nishikawa et al. developed a Pb-free solder with a composition of Sn-45Bi-2.6Zn and found similar advantages in Zn segregation at the Sn/Bi phase boundary to inhibit the interdiffusion of Sn and Bi atoms [21,22]. Ho et al. studied the effect of Zn addition on the corrosion performance of Sn-Bi solder and verified that Zn addition deteriorated the corrosion resistance of the solder [23]. Zhang et al. confirmed that adding minor Zn into Sn-10Bi increased the electromigration reliability of solder joints [24].

Besides Cu substrates, electroless nickel-immersion gold (ENIG, Ni/Au) is also commonly used for surface finishes in the electronics industry. ENIG provides a better antioxidant performance compared with the Cu surface finish [25]. In this paper, we tried to incorporate 0.2 or 0.5 wt.% Zn into Sn-10Bi solder and investigated the effect of Zn dopant on the compositional changes on the interfacial IMC layer between Sn-10Bi-based solder and Ni/Au surface finish, on the IMC growth in solder joints, and on the shear properties of solder joints after soldering and aging.

## 2. Materials and Methods

Sn-10Bi low-Bi-content solder with a 0.2 or 0.5 wt.% Zn dopant was selected in this paper. High-purity Sn, Bi, and Zn were accurately weighed and sealed in a quartz tube with a vacuum-sealing machine. The solder alloy was mixed at 600 °C for 60 min.

**Figure 1:**
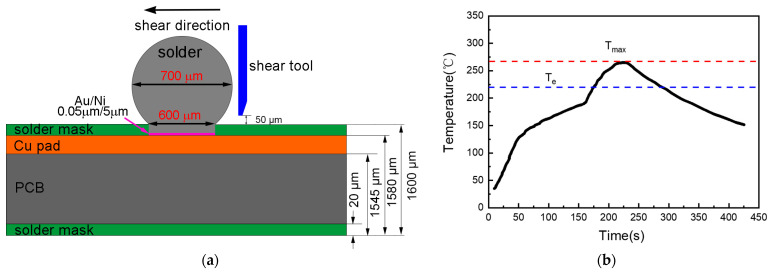
(**a**) Sketch of the PCB, solder ball, and shear testing setup; (**b**) Reflow temperature profile.

Solder joints were prepared with solder balls on an ENIG surface finish. To prepare the solder balls, the casted solder alloys were mechanically rolled into foils with a thickness of 35 µm and then punched into disks with a 3 mm diameter by utilizing a disk-punch system. These solder disks were then melted and recast into solder balls in the molten rosin. The measured diameter of these solder balls was kept at about 0.7 mm. We designed a tested printed circuit board (PCB) with Ni/Au as the surface finish on a Cu pad. The details of the PCB are illustrated in Figure 1a. Before soldering, the solder paste flux (NC-218-ASM no-clean flux, AMTECH Inc., Shenzhen, China) was deposited on the pads with the paste stenciling method, and then solder balls were positioned on the Ni/Au pads. The soldering process was completed with the temperature profile shown in Figure 1b. The melting point of Sn-10Bi solder is 219.4 °C [26], and so, accordingly, the peak temperature was set at about 260 °C. After soldering, the solder joints were aged under high-temperature conditions, including temperatures of 130 °C and 170 °C, and the duration of the aging period was 10–40 days.

Cross-sectioned samples were then mounted into conductive resin and polished to a 0.05 μm final finish with a colloidal silica solution. The interfacial microstructure of the solder joints was observed with a scanning electron microscope (SEM), and the interfacial composition was detected using an energy-dispersive spectrometer (EDS). Meanwhile, the lattice structure of the interfacial product was calculated via first-principles theory to confirm the interfacial composition. The mean thickness of the interfacial IMC layer was calculated using the “area by length” method.

To assess the impact of Zn dopants in Sn-10Bi solder on the properties of solder joints, shear tests were conducted on both as-soldered and as-aged joints. Figure 1a also depicts how the shear tests were conducted. All of the shear tests were finished at a shear speed of 0.1 mm/s. An SEM was used to observe the fracture morphologies.

## 3. Results and Discussion

### 3.1. Effect of Zn on Sn-Bi/Au/Ni Interfacial Reaction during Soldering

**Figure 2:**
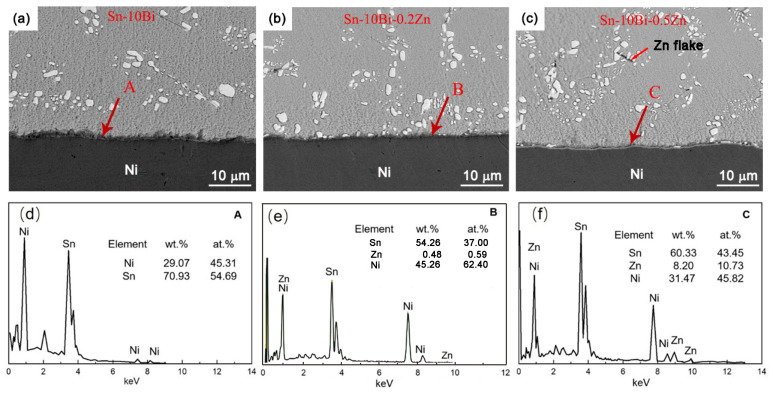
SEM images of the interfacial structure and the corresponding elemental compositions: (**a**,**d**) Sn-10Bi/Au/Ni, (**b**,**e**) Sn-10Bi-0.2Zn/Au/Ni, and (**c**,**f**) Sn-10Bi-0.5Zn/Au/Ni. The EDS results were obtained from the interfacial area marked with A, B and C from SEM images.

Figure 2 displays the SEM images of the interfacial structure between the Sn-10Bi-xZn solder and Ni/Au surface finish after soldering. In the solder matrix, the bright and gray areas are Bi and Sn phases, respectively. In this study, the thickness of immersion gold on the PCB was about 0.05 μm, and it was completely dissolved into the solder matrix during soldering. Regarding the existence of Zn phases in solder matrices, some researchers [27,28,29] have observed Zn-rich phases with granules or rods in Sn-Bi-Zn alloys with a higher Zn content. Meanwhile, according to the literature [30], the maximum solubility of Zn in Sn is about 0.34 wt.%. In Figure 2, Zn-rich flakes can be observed in the microstructure of the Sn-10Bi-0.5Zn solder matrix. Similar flakes have also been observed in Sn-58Bi solder with the addition of 0.5Zn or 1.0Zn [29].

During soldering, the Au layer was quickly dissolved into the molten solder, and can thus be neglected. The following results and discussion are mainly based on the reaction between the solder and Ni substrate. Ni atoms dissolved into the Sn-10Bi solder from the Ni substrate, while Sn atoms diffused into the Ni substrate from the molten solder. The area near the Ni substrate was gradually supersaturated with Ni due to the continuous dissolution of Ni atoms, which resulted in the formation of an IMC at the interface. Figure 2d–f show the EDS elemental results for the interfacial IMC layer from areas A, B, and C. As depicted in Figure 2d, the IMC layer from region A at the Sn-10Bi/Ni interface consisted of Sn and Ni atoms with atomic ratios approximately following 4:3. Thus, it is identified as Ni_3_Sn_4_, aligning with previous experimental evidence [22]. The formation of the Ni_3_Sn_4_ IMC primarily occurred through the grain boundary diffusion of Ni and grain coarsening within the IMC layer [31]. In the Zn-containing solder joints, as shown in Figure 2e,f, the IMC layers labeled B and C at the Sn-10Bi-Zn/Au/Ni interfaces were composed of Ni-Zn-Sn IMCs.

**Figure 3:**
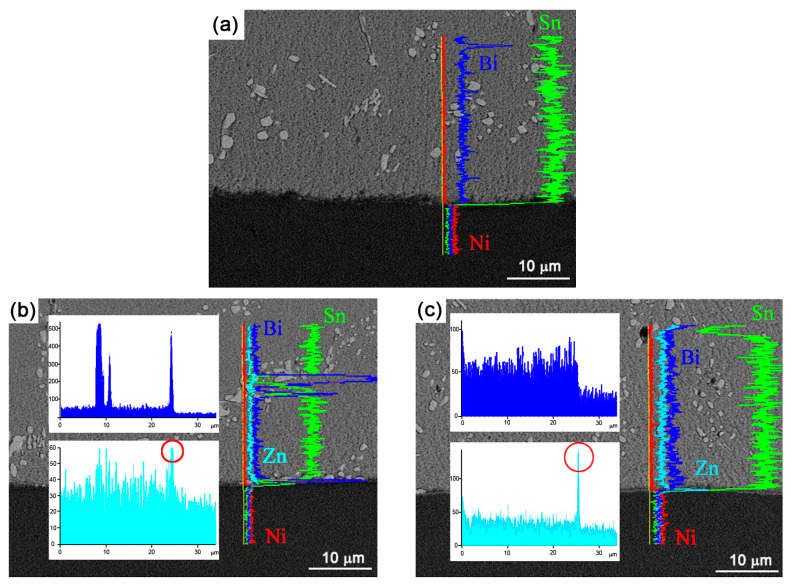
Elemental line analysis at the interface of (**a**) Sn-10Bi/Au/Ni, (**b**) Sn-10Bi-0.2Zn/Au/Ni, and (**c**) Sn-10Bi-0.5Zn/Au/Ni. Zn accumulation was observed in the Zn-containing solder joints and is marked with red circles in these images.

To investigate the distribution of elements at the interface of Sn-10Bi-xZn/Au/Ni solder joints, an elemental line analysis was conducted, with the results being shown in Figure 3. In the Sn-10Bi solder joint, the microstructure was mainly composed of Bi-rich and β-Sn phases. Bi atoms were dissolved in Sn phases, and played the solid-solution-strengthening effect [32]. During the interfacial reaction, the predominant diffusion atoms were Ni atoms from the substrate and Sn atoms from the solder. The ongoing dissolution of Ni led it to react with Sn to form Ni_3_Sn_4_:

3Ni + 4Sn → Ni_3_Sn_4_ (1)

After Zn dopant, the elemental line analysis revealed a peak value of Zn atoms at the interface, as shown in Figure 3b,c, marked with a red circle. During the interfacial reaction between Sn-10Bi-Zn and Ni, Zn atoms diffused into the interfacial region and reacted with the Sn and Ni atoms to form Ni_3_(Sn, Zn)_4_:

3Ni + 4(Sn, Zn) → Ni_3_(Sn, Zn)_4_ (2)

### 3.2. Effect of Zn on Sn-Bi/Au/Ni Interfacial Evolution during Isothermal Aging

**Figure 4:**
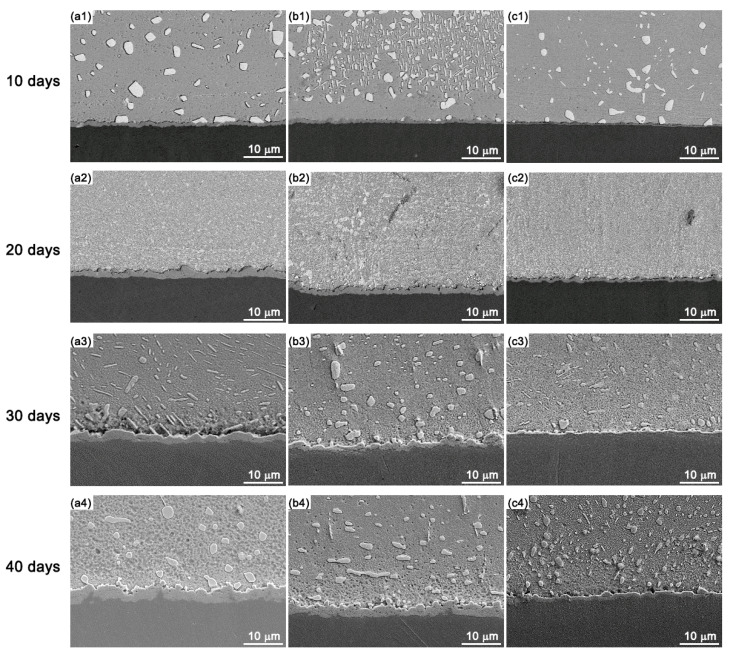
IMC growth in Sn-10Bi-Zn/Au/Ni during thermal aging at 130 °C: (**a1**–**a4**) Sn-10Bi, (**b1**–**b4**) Sn-Bi-0.2Zn, and (**c1**–**c4**) Sn-Bi-0.5Zn.

In order to study the IMC growth in Sn-10Bi-Zn/Au/Ni solder joints, the specimens were aged at temperatures of 130 °C and 170 °C for different amounts of days to study the effects of aging temperature and aging period duration on the interfacial evolution. The interfacial structures observed at the temperature of 130 °C are shown in Figure 4. After 40 days, it was found that the Zn dopant had helped to refine the microstructure in the Sn-Bi solder matrix, which can be explained by the accumulation of Zn atoms at the Sn-Bi phase boundaries. The Zn atoms served as barriers to prevent the interdiffusion of Sn and Bi atoms through the grain boundaries [29]. The refined microstructure helped to increase the strength of solder matrix, and then affected the fracture mode during the ball shear tests. In the Sn-Bi-Zn/Au/Ni solder joints, the interfacial IMC thickness increased with the amount of aging days. Moreover, the IMC layers in solder joints without a Zn dopant were obviously thicker than those in solder joints with a Zn dopant at equivalent aging durations, which illustrated that the interfacial growth was suppressed by the Zn dopant during the high-temperature aging process.

**Figure 5:**
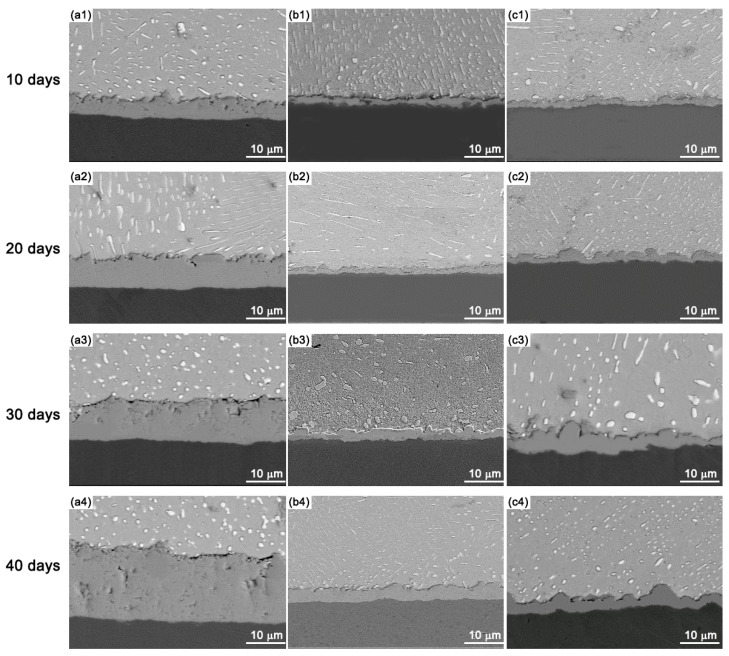
IMC growth in Sn-Bi-Zn/Au/Ni during thermal aging at 170 °C: (**a1**–**a4**) Sn-10Bi, (**b1**–**b4**) Sn-Bi-0.2Zn, and (**c1**–**c4**) Sn-Bi-0.5Zn.

To further investigate the effect of aging temperature on the interfacial evolution and observe the inhibited effect of Zn dopant addition on IMC growth, the specimens were aged at a high temperature of 170 °C, and the results of this are shown in Figure 5. The uneven distributed Ni_3_Sn_4_ at the interface of Sn-Bi/Au/Ni became flattened during aging, while the shape of Ni_3_(Sn, Zn)_4_ at the Sn-Bi-Zn/Au/Ni interface did not change much. Usually, the interfacial IMC was found to have a scallop-shaped morphology after soldering. During the high-temperature aging, the scallop-shaped IMC was flattened because the grooves along the scallop structure provided the shortest path for Sn and Ni atoms during diffusion. Also, the transition from a scalloped shape to a flattened shape was beneficial for decreasing the surface-to-volume ratio of the IMC grain and its interfacial energy. A longer aging period or a higher aging temperature promoted the flattening of the IMC layer. Compared with Figure 4, it is evident that an increase in temperature increased the interfacial reaction process and accelerated the rate of IMC formation. During the isothermal aging process, there was a quicker interdiffusion between Ni and Sn atoms. As a result, the Ni_3_Sn_4_ layer at the Sn-Bi/Au/Ni interface significantly increased in thickness during aging. For solder joints with a Zn dopant, the growth of the Ni_3_(Sn, Zn)_4_ layer was slower, which also postponed the flattening process in the IMC layer.

It seems that Zn dopant addition can inhibit the IMC growth at the joints of Sn-10Bi solder with a Ni/Au surface finish. Regarding the effect of Zn addition into solder on the interfacial IMC composition, studies [33,34] have investigated the interfacial reaction of Sn-58Bi solder with the incorporation of 0.7 wt.% Zn on a copper substrate, and identified a CuZn IMC formation over an interfacial Cu_6_(Sn, Zn)_5_ layer at the interface. In our pervious study [35], we examined the interface of Sn-10Bi solder with a 0.5 wt.% Zn dopant on a copper substrate and observed the formation of a Cu_5_Zn_8_ IMC during soldering, and then Cu_6_(Sn, Zn)_5_ under high-temperature aging. In contrast, Ni_3_Sn_4_ and Ni-Sn-Zn products generated by the reaction between Sn-10Bi-xZn solder and a Ni/Au surface finish seem to be more stable and will not decompose during high-temperature aging.

**Figure 6:**
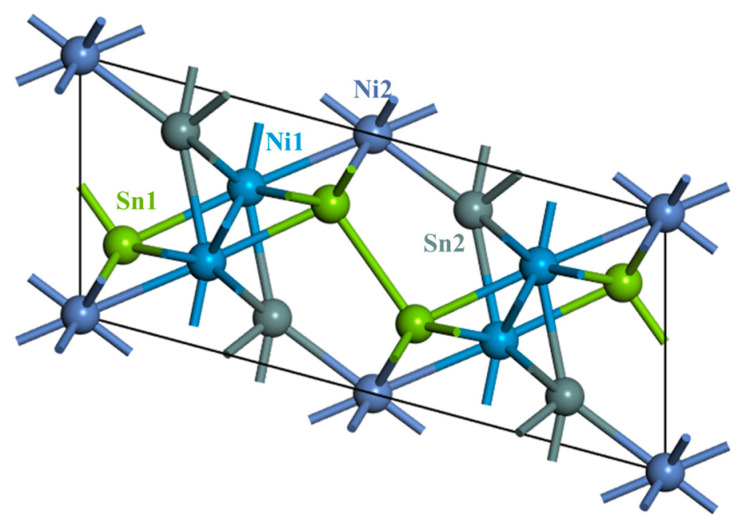
Schematic of Ni_3_Sn_4_. The atoms in different colors belong to different Wyckoff positions.

Calculations based on first-principles theory were carried out to confirm the detailed crystal structure of the Ni-Sn-Zn IMCs. All calculations were performed in the Vienna Ab initio Simulation Package (VASP 6.4.3) [36] with the help of VASPKIT [37]. Electron–ion interactions were described by projector augmented wave (PAW) pseudopotentials [38]. The exchange–correlation functional was chosen as the Perdew–Burke–Ernzerhof functional within the framework of generalized gradient approximation (PBE-GGA) [39]. The Brillouin-zone sampling was described by the Monkhorst–Pack scheme [40], with a grid mesh of 4 × 12 × 10. The plane-wave cutoff energy was set as 520 eV. Figure 6 shows the crystal structure of the Ni_3_Sn_4_ phase, with the space group of Ni_3_Sn_4_ belonging to C2/m. There are four inequivalent Wyckoff positions in Ni_3_Sn_4_: Ni1: 4i (0.2158, 0, 0.3362), Ni2: 2a (0, 0, 0), Sn1: 4i (0.0725, 0.5, 0.3130), and Sn2: 4i (0.3283, 0.0, 0.8177). For a Ni-Sn-Zn IMC, there are four possible sites, with Zn substituting a Sn2, Sn1, Ni1, or Ni2 in the crystal, as listed in Table 1. The calculated results for Ni_3_Sn_4_ are consistent with those reported in the literature, which confirms the suitability of our selection of parameters. The formation energies of the IMCs were calculated in order to determine their thermodynamic stability, and it seems that more negative formation energy was required with Sn sites in Ni_3_Sn_4_ crystals substituted by Zn atoms when compared with Ni sites substituted by Zn atoms. Zn atoms preferred to occupy the Sn2 site to create a stable IMC crystal, namely Ni_3_(Sn_3.5_Zn_0.5_) or Ni_3_(Sn, Zn)_4_.

**Figure 7:**
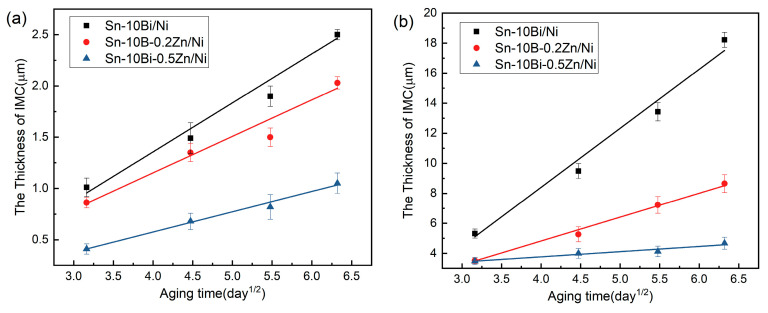
The rate of IMC growth in Sn-Bi-Zn/Au/Ni after aging at different temperatures: (**a**) 130 °C and (**b**) 170 °C.

IMC formation in solder joints is very important for the reliability of electronic interconnects and affects the lifetime of electronic packaging systems. In Sn-10Bi-Zn/Au/Ni solder joints, the thickness of interfacial IMC layers increased with the amount of isothermal aging, potentially deteriorating the mechanical reliability of the electronic connections. The growth of IMCs during high-temperature aging is mainly diffusion-controlled, and the thickness of the IMC (*X*) can be described using the following equation:

(3) $$X=X_{0}+\sqrt{Dt}$$

where *X*_0_ is the initial thickness of the IMC, *D* is the diffusivity, and *t* is the aging time. Figure 7 plots the relationship between IMC thickness and aging time at the aging temperatures of 130 °C and 170 °C. There is a linear relationship between IMC thickness and the square root of aging time.

The growth rate ($\sqrt{D}$) during aging can be derived from the linear fit to the curves shown in Figure 7, illustrating that the Zn dopant modified the IMC’s formation and growth. According to Figure 7a, it is found that the IMC’s thickness in Sn-Bi/Au/Ni joint increased from 1.01 μm in the as-soldered condition to 2.51 μm after 40 days of aging, and the growth rate was calculated to be 0.47 µm/day^1/2^. At the interface, the activity of Ni and Sn atoms increased during aging at 130 °C to promote the formation of Ni_3_Sn_4_, and thus the IMC thickness increased progressively with time. With an aging period of up to 40 days, the IMC thickness in the 0.2Zn-containing solder joint increased from 0.86 μm to 2.03 μm, and that in the 0.5Zn-containing solder joint only increased from 0.41 μm to 1.05 μm. There was a notable decrease in the IMC thickness in the 0.5Zn-containing solder joint compared to those without Zn or with 0.2 Zn. The IMC growth rate was calculated to be 0.35 µm/day^1/2^ for Sn-10Bi-0.2Zn/Au/Ni and 0.19 µm/day^1/2^ for Sn-10Bi-0.5Zn/Au/Ni. As shown in Figure 7b, a higher aging temperature further promoted the fast growth of the interfacial IMC layer. The IMC growth rate in solder joints aged at 170 °C was 3.92 μm/day^1/2^, 1.59 μm/day^1/2^, and 0.35 μm/day^1/2^, respectively. Comparing it with our previous experiments [35], we find that the IMC growth rate on a Ni/Au surface is lower than that on a Cu surface finish. Doping with trace Zn seems to enhance the thermal stability of the Sn-10Bi-Zn/Au/Ni interface. In a paper by Qi et al. [45], the growth rate of an interfacial IMC in Sn-3.5Ag-0.5Cu solder on a Ni substrate was reported to be about 0.25 μm/day^1/2^ during isothermal aging at 125 °C. Therefore, the growth rate of IMC in our Sn-10Bi/Ni joints was little higher than that observed in Sn-Ag-Cu Pb-free solder joints, and with 0.5 wt.% Zn dopant into Sn-10Bi solder, the IMC presented a lower growth rate compared with Sn-Ag-Cu solder joints.

### 3.3. Effect of Zn on the Shear Properties of Sn-Bi/Au/Ni Joints

The excellent mechanical properties of solder interconnects greatly ensure the reliability of electronic products. The mechanical characteristics of Sn-10Bi-Zn solder joints on a Ni/Au surface finish were evaluated through shear tests. Statistical analyses were conducted on the shear strength of the as-soldered and as-aged joints, and the fracture mode was evaluated in order to determine the effect of Zn dopants on solder joints.

**Figure 8:**
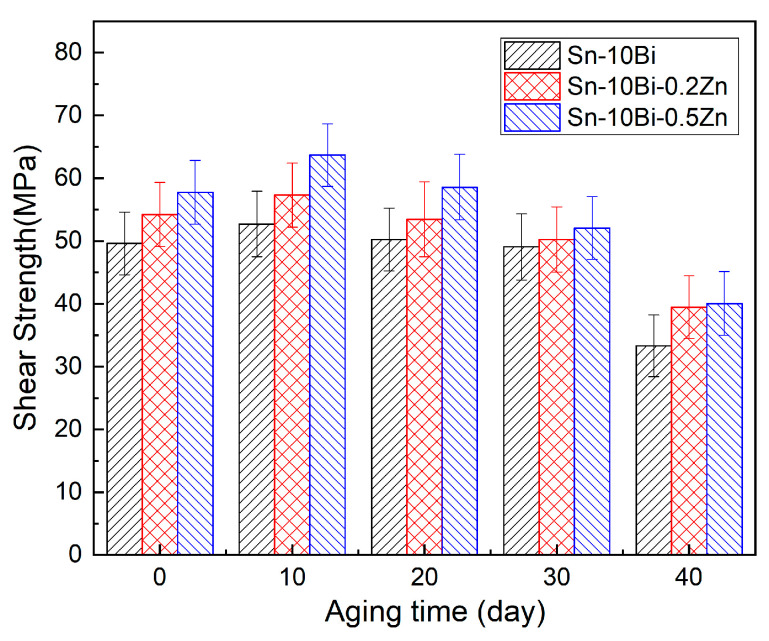
Shear strength of solder joints aged at 130 °C.

For this paper, we selected as-soldered joints and as-aged joints that had been processed at 130 °C for the shear tests. The shear curves are provided in the Supplementary Materials. Figure 8 shows the shear strength from the shear curves, including the changes in shear strength with different solder compositions and aging durations. The aging time notably impacted the shear strength of solder joints, with the shear strength of Sn-10Bi-Zn solder joints initially increasing and subsequently decreasing as the aging time progressed. This observation parallels the findings by Ahmed et al. [46]. The average strength of the as-soldered joints with 0, 0.2, and 0.5 wt.% Zn is about 40 MPa, 44 MPa, and 47 MPa, respectively, which illustrates that Zn dopant addition will be helpful for improving the joint strength of Sn-10Bi solder joints. After aging for 10 days, the joint strength increased to about 52 MPa, 57 MPa, and 63 MPa, respectively. However, as the amount of aging days increased and the IMC thickness grew, the adhesive strength between the solder and surface finish was impacted, causing a decrease in shear strength to 33 MPa, 39 MPa, and 41 MPa, respectively. With the same amount of aging days, the Zn dopant also helped to increase the joint strength compared to Sn-10Bi-Zn with Sn-10Bi solder joints. As seen in Figure 8, the high-temperature aging had the greatest effect on the shear strength of the solder joints, because it promoted rapid IMC growth, which consequently deteriorated the interfacial bonding between the solder and substrate. On the other hand, Zn addition inhibited the interfacial IMC growth in solder joints, and therefore Zn-doped solder joints always presented a higher shear resistance compared with Sn-10Bi solder joints. According to a paper by Tsukamoto et al. [47], the ball shear strength of Sn-3.0Ag-0.5Cu solder on a Ni(P)/Au surface finish after soldering and aging for 1000 h (41.67 days) at 125 °C was about 61 MPa and 51 MPa, respectively. Therefore, Sn-10Bi-0.5Zn solder can provide a similar shear performance to Sn-3.0Ag-0.5Cu solder on a Ni/Au surface finish.

**Figure 9:**
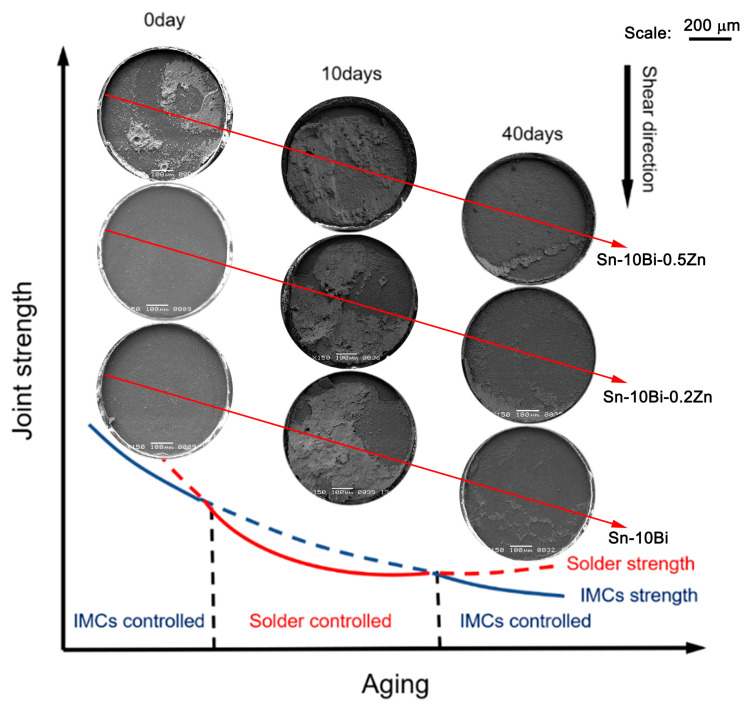
Schematic of the joint strength, either determined by the solder itself or by the interfacial IMC, and its relationship with the fracture morphology for Sn-10Bi-Zn/Au/Ni solder joints.

During the shear tests on ball solder joints, the shear strength was controlled by the competition between the solder strength and the interfacial bonding force. Examining the fracture morphologies is beneficial for understanding the crack propagation and failure modes. Figure 9 shows the evolution of fracture morphologies for Sn-10Bi-Zn/Au/Ni solder joints with different aging durations and a schematic illustrating the fracture mode when determined by the solder itself or by the interfacial IMC. When the shear resistance of the solder ball exceeded the interfacial bonding, a brittle fracture mode possibly occurred at the interface between the solder and the Ni/Au surface finish. Conversely, a ductile failure mode occurred with fracturing in the solder matrix. Additionally, there existed a quasi-ductile or quasi-brittle failure mode, which mostly occurred with fractures in the solder or along the interface.

In Figure 9, the brittle mode can be deduced from the fracture morphologies of the as-soldered joints. In the solder matrix, Zn improved the strength of the solder matrix and Bi played a solution-strengthening role, which caused the solder strength to be higher than the interfacial bonding force. Previous studies have indicated that adding Zn can refine the microstructure and enhance the mechanical properties of solder alloys [22]. Accordingly, failure occurred along the interface with a brittle fracture mode. In fact, the brittle characteristic of these Sn-10Bi-Zn/Au/Ni solder joints also stemmed from the low dissolution rate of Ni in Sn-based solder during the soldering process [48,49], which induced a reduction in the IMC’s thickness and the interfacial bonding strength.

After aging for 10 days, the fracture mode transitioned from brittle to ductile or quasi-ductile, which means that the solder strength decreased and the interfacial bonding strength increased. Moreover, an increase in the IMC thickness with a shorter aging duration helped to improve the interfacial bonding strength, while the coarsening of the solder microstructure decreased the solder strength. Accordingly, the competition between the solder strength and interfacial bonding decided the failure within the solder matrix.

As the aging time further increased, the brittle fracture mode occurred again with fractures along the interface. The IMC layer thickened with prolonged aging, which decreased the interfacial bonding strength. Throughout the isothermal aging process, the growth rate of the Ni_3_Sn_4_ layer surpassed that of the Ni_3_(Sn, Zn)_4_ layer, leading to a lower shear strength for Sn-10Bi/Au/Ni solder joints compared to Sn-10Bi-Zn/Ni joints after 30 to 40 days of aging.

## 4. Conclusions

In this paper, the bonding of Sn-10Bi with a minor Zn dopant on a Ni/Au surface finish was studied based on the interfacial composition, IMC growth, and shear strength under isothermal aging conditions. Our main conclusions are as follows:

(1) During soldering, the interfacial IMC layer in Sn-10Bi/Au/Ni solder joints was determined to be Ni_3_Sn_4_. With a 0.2 or 0.5 Zn dopant, Zn atoms were enrolled in the interfacial reaction to form a Ni-Sn-Zn IMC at the interface. Calculations via first-principles theory confirmed that it was a more stable Ni_3_(Sn, Zn)_4_ IMC structure because the Sn atoms in the Ni_3_Sn_4_ crystal had been substituted by Zn atoms.
(2) Adding a Zn dopant into Sn-10Bi solder effectively inhibited the growth of IMCs in solder joints on a Ni/Au surface finish during high-temperature aging.
(3) Adding a Zn dopant into Sn-10Bi solder increased the shear strength of Sn-10Bi/Au/Ni solder joints. The joint strength reached its maximum at about 65 MPa for Sn-10Bi-0.5Zn solder joints after isothermal aging for 10 days, which is similar to the result reported for Sn-3.0Ag-0.5Cu solder.
(4) The fracture mode was controlled by the Zn addition and the duration of aging, with the fracture mode changing from brittle in the as-soldered condition, to quasi-ductile after 10 days of aging, and again to brittle after a long period of aging, which reflected the competition between solder strength and IMC growth.

## Figures and Tables

**Table 1:** Lattice parameters and formation energy of Ni_3_Sn_4_ and Zn-doped Ni_3_Sn_4_.

	a (Å)	b (Å)	c (Å)	β (°)	Volume (Å^3^)	Ef (eV/atom)
Ni_3_Sn_4_	12.301	4.101	5.275	105.1	256.91	−0.2740
Sn2: Ni_3_(Sn_3.5_Zn_0.5_)	12.196	4.098	5.224	106.06	250.89	−0.2480
Sn1: Ni_3_(Sn_3.5_Zn_0.5_)	12.242	4.086	5.221	106.53	250.33	−0.2451
Ni1: (Ni_2.5_Zn_0.5_)Sn_4_	12.467	4.125	5.366	105.26	266.21	−0.2107
Ni2: (Ni_2.5_Zn_0.5_)Sn_4_	12.455	4.211	5.303	105.69	267.82	−0.2097
Ni_3_Sn_4_ [41]	12.29	4.1	5.27	-	256.2	−0.287
Ni_3_Sn_4_ [42]	12.345	4.111	5.323	105.5	-	−0.316
Ni_3_Sn_4_ [43]	12.537	3.889	5.272	-	257.46	−0.234
Ni_3_Sn_4_ [44]	12.418	4.111	5.315	105.48	-	−0.267

## Data Availability

The original contributions presented in this study are included in the article; any further inquiries can be directed to the corresponding authors.

## Supplementary Materials

The following supporting information can be downloaded at https://www.mdpi.com/article/10.3390/ma17174364/s1, Figure S1: Shear curve of unaged solder joints; Figure S2: Shear curve of solder joints aged at 130 °C for 10 days; Figure S3: Shear curve of solder joints aged at 130 °C for 20 days; Figure S4: Shear curve of solder joints aged at 130 °C for 30 days; Figure S5: Shear curve of solder joints aged at 130 °C for 40 days.
