# Supplementary material for: Effects of Minor Zn Dopants in Sn-10Bi Solder on Interfacial Reaction and Shear Properties of Solder on Ni/Au Surface Finish
Source: Materials (Basel). 2024 Sep 3;17(17):4364. doi: 10.3390/ma17174364 (PMC11396524; doi:10.3390/ma17174364)
Supplement: Supplementary file 1 [file materials-17-04364-s001.zip › materials-3089553-supplementary.pdf]

Shear data on solder joints:

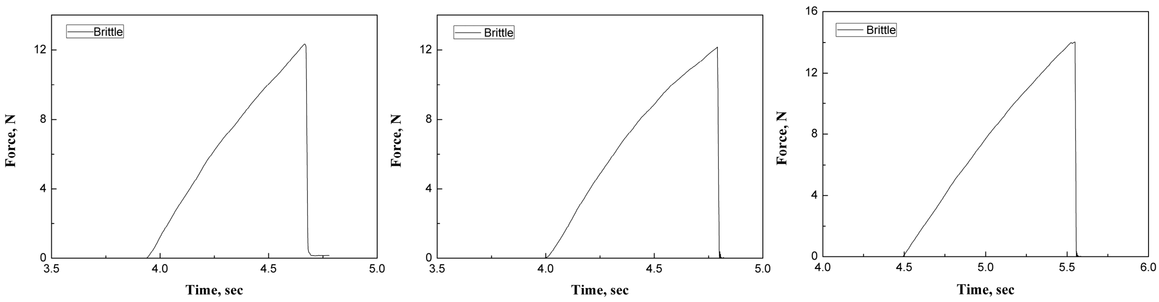

(a)Sn-10Bi/Ni (b)Sn-10Bi-0.2Zn/Ni (c)Sn-10Bi-0.5Zn/Ni

Figure S1 Shear curve of unaged solder joints

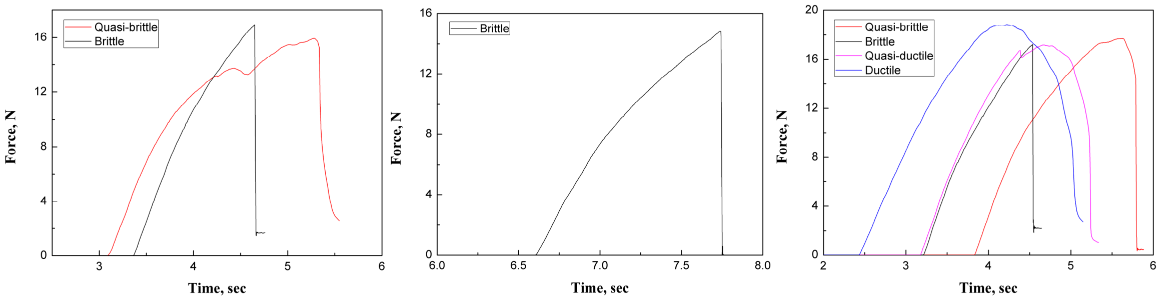

(a)Sn-10Bi/Ni (b)Sn-10Bi-0.2Zn/Ni (c)Sn-10Bi-0.5Zn/Ni

Figure S2 Shear curve of solder joints aged at 130℃ for 10 days

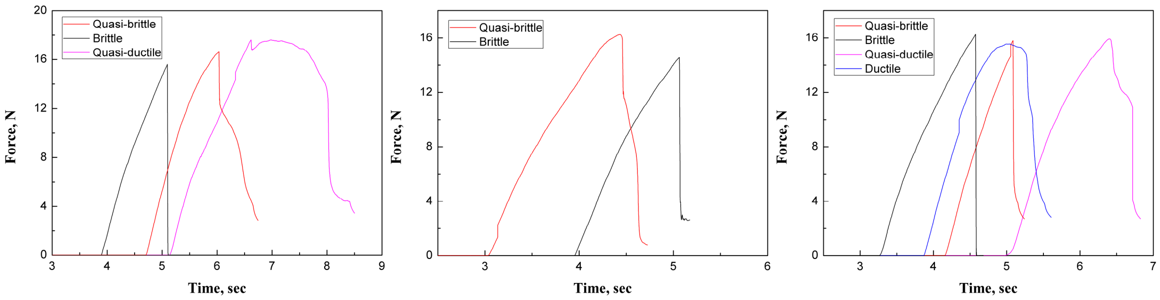

(a)Sn-10Bi/Ni (b)Sn-10Bi-0.2Zn/Ni (c)Sn-10Bi-0.5Zn/Ni

Figure S3 Shear curve of solder joints aged at 130℃ for 20 days

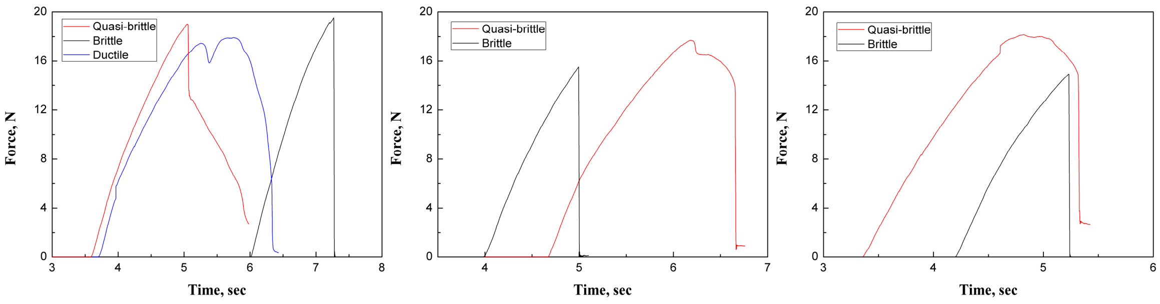

(a)Sn-10Bi/Ni (b)Sn-10Bi-0.2Zn/Ni (c)Sn-10Bi-0.5Zn/Ni

Figure S4 Shear curve of solder joints aged at 130℃ for 30 days

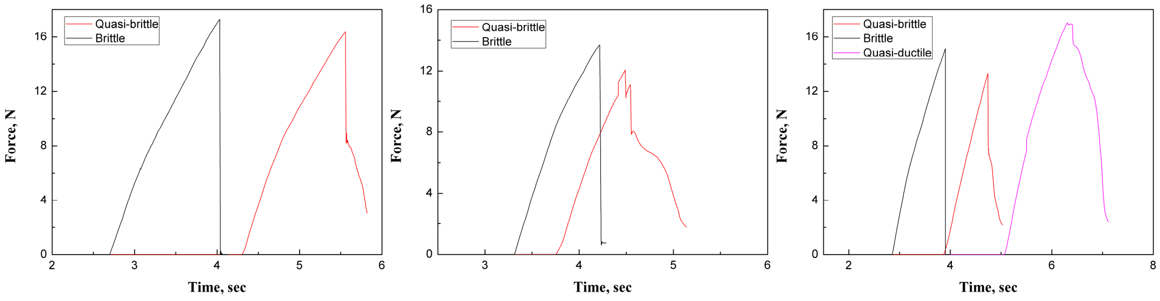

(a)Sn-10Bi/Ni (b)Sn-10Bi-0.2Zn/Ni (c)Sn-10Bi-0.5Zn/Ni

Figure S5 Shear curve of solder joints aged at 130℃ for 40 days
